# Proceedings of the Hom. Med. Society of Ohio

**Published:** 1882-01

**Authors:** 


PROCEEDINGS of the HOMŒOPATHIC MEDICAL SOCIETY of OHIO. Seventeenth Annual Session. Pp. 174. Advance Printing Co.: Cincinnati. 1881.

The meeting of this prosperous Society was held at Toledo, in May, 1881. The secretary, Dr. Beebe, has presented his colleagues with a very neat and interesting volume. Reports were presented from the bureaus of materia medica, physiology and pathology, obstetrics, gynæcology, otology, etc., insanity, surgery, clinical medicine, and sanitary science. We wish the Society every success.

